# *Glabrous Rice 1*, encoding a homeodomain protein, regulates trichome development in rice

**DOI:** 10.1186/1939-8433-5-32

**Published:** 2012-10-06

**Authors:** Jinjun Li, Yundong Yuan, Zefu Lu, Liusha Yang, Rongcun Gao, Jingen Lu, Jiayang Li, Guosheng Xiong

**Affiliations:** Jiaxing Academy of Agricultural Sciences, Jiaxing, Zhejiang Province 314016 China; State Key Laboratory of Plant Genomics and National Center for Plant Gene Research, Institute of Genetics and Developmental Biology, Chinese Academy of Sciences, Beijing, 100101 China

**Keywords:** Glabrous rice, Trichome development, WOX protein

## Abstract

**Background:**

Glabrous rice, which lacks trichomes on the rice epidermis, is regarded as an important germplasm resource in rice breeding. Trichomes are derived from aerial epidermal cells and used as a model to study the cell fate determination in plant. In Arabidopsis, the molecular mechanisms of trichome development have been well studied. However, little is known about the molecular basis of trichome development in rice.

**Results:**

In this study, near isogenic lines harboring the *glabrous rice 1* locus were developed. By a map-based approach, we narrowed down the locus to a 21-kb DNA region harboring two genes. One of the genes named *Glabrous Rice 1* (*GLR1*), which is most likely the candidate, encodes a homeodomain protein containing the WOX motif. Constitutive Expression of *GLR1* could partially complement the glabrous phenotype of NIL^*glr1*^. The knock down of *GLR1* by RNA interference led to a significant decrease in trichome number on the leaves and glumes of the RNAi transgenic plants.

**Conclusion:**

*GLR1* plays an important role in rice trichome development and will contribute to breeding of glabrous elite rice varieties.

**Electronic supplementary material:**

The online version of this article (doi:10.1186/1939-8433-5-32) contains supplementary material, which is available to authorized users.

## Background

The glabrous feature of rice is considered as a favorite agronomic trait for rice farmers because it has greater packing capability of rice grains and produces less dust that causes itching effect on farmers. Glabrous rice lacks trichomes on leaves and glumes (Khush*, et al.*^2001^). Most rice cultivars in America are glabrous and recognized as an important germplasm resource in breeding due to its high yield, good quality, and wide compatibility in crossing with other rice varieties (Guo*et al.*^1999^, Luo *et al.*^2000^). Trichomes are derived from aerial epidermal cells and serve various protective purposes such as insect herbivore resistance, freezing tolerance, and shade of UV irradiation (Ishida et al. 2008). There are two distinct types of trichomes developed on leaves of monocot plants. One is macrohairs on silica cells, the other is microhairs along the stomata cells (Khush, et al. 2001). So far, a number of glabrous mutants have been identified in many plant species, including *Arabidopsis,* tomato, cotton, and maize (Machado *et al.*^2009^, Moose *et al.*^2004^, Rerie *et al.*^1994^, Yang *et al.*^2011^). However, the molecular mechanism underlying trichome development has only been intensively investigated in *Arabidopsis.*

In *Arabidopsis*, trichome development has been used as a model system to study the cell fate determination and shown to be regulated by a complex gene network (Ishida *et al.*^2008^). A homeodomain-leucine zipper protein *GLABRA2* (*GL2*) and an R3 Myb protein *TRIPTYCHON* (*TRY*) play essential roles in trichome initiation and hairless cell differentiation (Rerie et al. 1994, Schellmann et al. 2002). The expression of *GL2* and *TRY* are regulated by the WD-repeat/bHLH/MYB complex including TRANSPARENT TESTA GLABRA1 (TTG1), GLABRA3 (GL3)/ENHANCER OF GLABRA3 (EGL3) and GLABRA1 (GL1). Epidermal cells expressing the GL2 protein are able to differentiate into trichome cells. The TRY protein expressed in trichome cells, however, can move into neighboring cells and compete with GL1 for binding to GL3/EGL3 to repress the *GL2* expression. The TRY mediated down regulation of the *GL2* expression inhibits trichome formation in neighboring cells (Ishida, et al. 2008). Actually, factors able to modulate this gene network affect the trichome development. Previous studies on mutants defective in the biosynthesis and/or signaling of gibberellins, salicylic acid, jasmonic acid, and cytokinin have showed that phytohormones are involved in trichome initiation (Gan *et al.*^2006^, Gan *et al.*2007b, Perazza *et al.*^1998^, Traw and Bergelson 2003, Zhou *et al.*^2011^). It has been turned out that roles of these phytohormones in trichome development are mediated by their effect on the expression or activity of the components of the WD-repeat/bHLH/MYB complexes. Roles of gibberellins and cytokinins in trichome initiation are mainly dependent on C2H2 transcription factors including GIS1, GIS2, ZFP5 and ZFP8. These transcription factors are able to promote the *GL1* expression (Gan *et al.*^2007^a, Maes *et al.*^2008^, Perazza*, et al.*^1998^, Zhou*, et al.*^2011^). In addition, JAZ proteins, the key components in the JA signaling pathway, have been shown to interact with bHLH transcription factors (GL3, EGL3 and TT8) and MYB transcription factors (MYB75 and GL1) (Qi *et al.*^2011^). The JA-induced destruction of JAZ proteins results in releasing the transcriptional function of the WD-repeat/bHLH/MYB complex and activating downstream events of trichome initiation. Furthermore, recent studies have shown that the microRNA156 targeted gene *SPL9* could bypass the function of *GL1* and directly binds to promoters of *TCL1* and *TRY* to activate their expression (Yu *et al.*^2010^).

In contrast to the sophisticated mechanisms revealed in *Arabidopsis*, little is known about the molecular mechanisms of trichome development in other plants. It has been noted that a couple of homeodomain-leucine zipper proteins, which are specifically expressed in epidermal cells, are essential in differentiation of epidermal cells. Outer Cell Layer 4 (OCL4), a maize HD-ZIP transcription factor, has been suggested to involve in the repression of macrohair differentiation (Vernoud, et al. 2009), and a HD-Zip protein in tomato, Woolly (Wo) that interacts with Cyclin B2, plays an essential role for trichome formation and embryonic development (Yang et al. 2011). In addition, another subfamily of the homeobox gene, known as *WUS-like homeobox* genes (*WOX*), may also play roles in division or differentiation of epidermal cells. *Pressed Flower* (*PRS*) is involved in activation of the proliferation of marginal cells. It has been observed that multicellular bulges with trichomes formed on stems and epidermal cells outgrow on sepals of *35S:PRS* transgenic plants (Matsumoto and Okada 2001). Moreover, *Narrow sheath 1* (*NS1*) and *Narrow sheath 2* (*NS2*), which are duplicated relatives of *PRS* in maize, have been suggested to play a role in a lateral domain of shoot apical meristems (Nardmann *et al.*^2004^). In addition, *OsWOX3* has been found to repress the expression of *OsYAB3*, which is required for cell differentiation during rice leaf development (Dai *et al.*^2007^).

Previous study showed that macrohairs on the leaf blade are greatly reduced in the maize *macrohairless 1* (*mhl1*) mutant (Moose*, et al.*^2004^). A major QTL controlling macrohairs in *Teosinte* has been found to locate near the maize gene *MHL1* (Lauter *et al.*^2004^). In rice, previous genetic analysis has identified a couple of loci that control trichome development. For example, *gl* regulates glabrous leaf and hull traits, *Hl*_*a*_ and *Hl*_*b*_ were related to long hair development on rice leaves and *Hg* may be responsible for the extreme long hairs on auricles and glumes (Nagao *et al.*^1960^). However, no gene controlling these traits has been cloned in rice as yet. Recently, *glabrous leaf and hull mutant* (*gl1*) has been reported to locate within a 54-kb region at the short arm of chromosome 5 (Li *et al.*^2010^, Wang *et al.*^2009^, Yu *et al.*^1995^), but the gene has not been identified yet. Here, we report the identification and characterization of the *Glabrous Rice 1* (*GLR1*), which controls the trichome development in rice. Our work extends an insight into the molecular mechanism of trichome development in rice. The identification and characterization of *GLR1* will facilitate breeders to develop elite glabrous rice varieties via marker-assisted-selection and genetic modification approaches.

## Results

### Phenotype of the near isogenic line of glabrous rice

**Figure 1:**
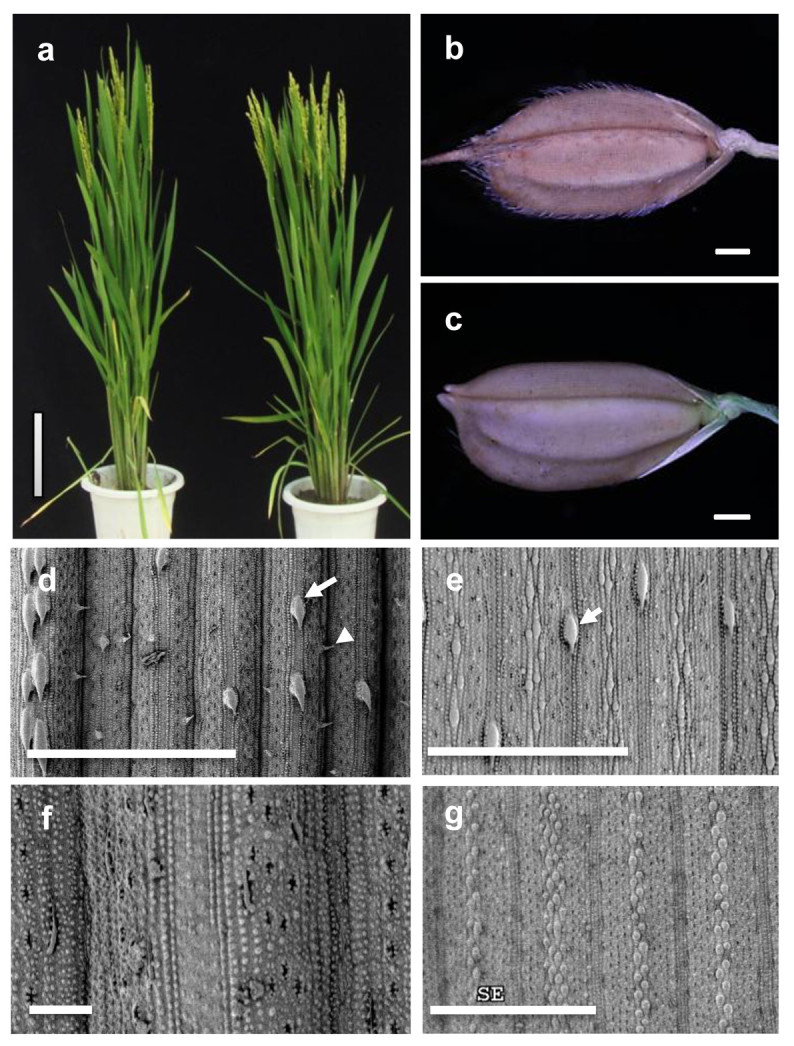
**Phenotypes of NIL**^***glr1***^**plant.** (**a**) Mature plants of NIL^*GLR1*^ (left) and NIL^*glr1*^ (right), Bar = 20 cm. (**b**, **c**) The grains of NIL^*GLR1*^ (**b**) and NIL^*glr1*^ (**c**) Bars = 0.1 cm. (**d** to **g**) The SEM views of the adaxial (**d**) and abaxial (**e**) sides of the NIL^*GLR1*^ leaves, and adaxial (**f**) and abaxial (**g**) sides of the NIL^*glr1*^ leaves . Arrow indicates the macrohair and arrowhead shows the microhair. Bars = 500 μm.

The glabrous variety Jia64 is derived from the American rice variety Rico No.1 and near isogenic lines (NIL) of glabrous rice developed by backcrossing Jia64 with a pubescent variety Jia33 for 5 generations. There are no obvious differences of the overall morphology between NIL^*GLR1*^ and NIL^*glr1*^ plants (Figure 1a). However, the leaves of NIL^*glr1*^ plants are smooth whereas leaves of NIL^*GLR1*^ plants are rough with many hairs. In contrast to glumes of the NIL^*GLR1*^ plant (Figure 1b), the glumes of the NIL^*glr1*^ plant showed no trichome or only a few trichomes growing on margins of the hull (Figure 1c). On rice leaves, there are two types of trichomes, macrohairs and microhairs. Scan Electronic Microscope (SEM) analysis showed that both macrohairs and microhairs on the abaxial and adaxial sides of NIL^*GLR1*^ leaves are able to be observed (Figure 1d and Figure 1e). However, neither macrohairs nor microhairs could be observed on both sides of NIL^*glr1*^ leaves (Figure 1f and Figure 1g).

### Map-based cloning of *GLR1*

**Figure 2:**
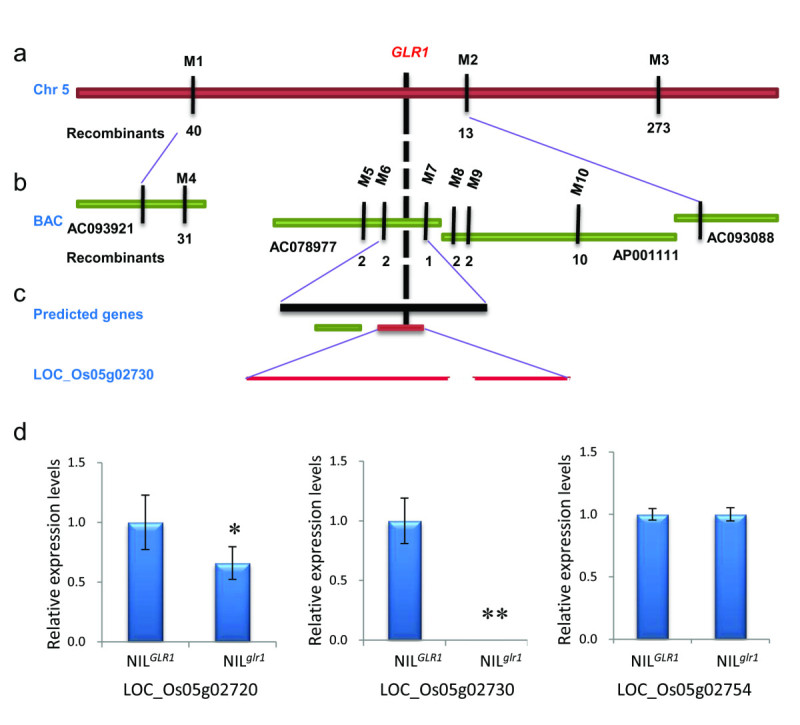
**Map-based cloning of** ***GLR1*** **.** (**a**) The *GLR1* locus was mapped in chromosome 5 between markers M1 and M2. Recombinants were identified from 1,447 F_2_ glabrous plants. (**b**) Fine mapping of the *GLR1* locus. The *GLR1* locus was narrowed to a 21-kb genomic DNA region between markers M6 and M7. (**c**) The *LOC_Os05g02720* (green) and *LOC_Os05g02730* (red) are predicted in the candidate region. The annotated gene of *LOC_Os05g02730* consists of two exons and one intron. (**d**) The relative expression levels of *LOC_Os05g02720*, *LOC_Os05g02730* and *LOC_Os05g02754* in young panicles of NIL^*GLR1*^ and NIL^*glr1*^ plants (T-test, P<0.05).

**Figure 3:**
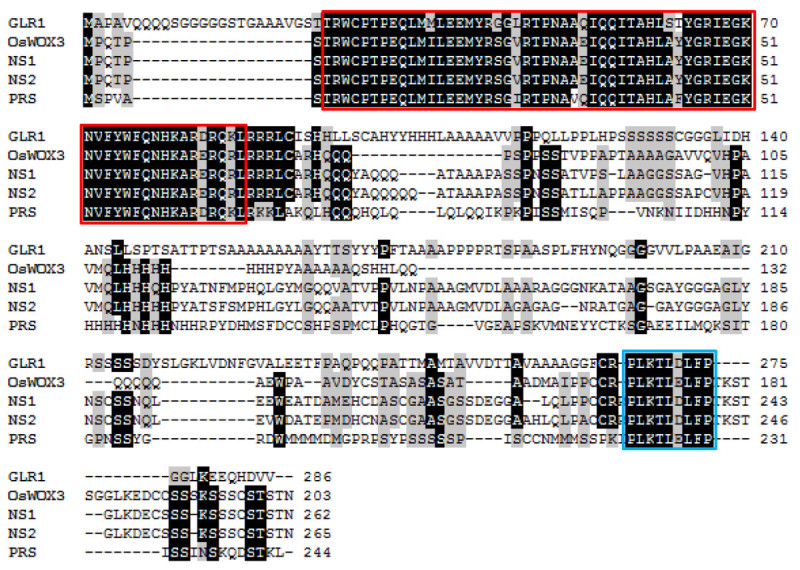
**GLR1 is highly homologous to the WOX3 subgroup proteins.** Alignment of rice GLR1, OsWOX3, *Arabidopsis* PRS, maize NS1 and NS2. Numbers at right refer to the positions of amino acid residues. The conserved homeodomain was indicated by the red box. The conserved WOX-box is indicated by blue box.

Previous genetic analysis has shown that the glabrous phenotype of America rice was controlled by a single recessive nuclear gene (Li *et al.*^1993^). To map the *GLR1* locus, an F_2_ mapping population was generated from a cross between Jia64 and a polymorphic *japonica* variety Jia33. Linkage analysis of 44 F_2_ plants having the glabrous phenotype showed that the *GLR1* locus located between the InDel marker M1 and the SSR marker M2 on chromosome 5 (Figure 2a and Table 1). This region is consistent with the previously mapped *gl1* locus on the short arm of chomsome 5 (Li, et al. 2010, Wang, et al. 2009). To fine-map *GLR1*, 1,447 F_2_ glabrous plants were analyzed using 7 newly developed markers (Figure 2b and Table 1) and *GLR1* was finally pin-pointed within an interval of 21-kb DNA fragment between the markers M6 and M7. Within this region, there are 2 predicted genes, *LOC_Os05g02720* (*Os05g0118600*) and *LOC_Os05g02730* (*Os05g0118700*) (Figure 2c). The former encodes a hypothetic protein and the latter encodes a homeobox-containing protein. Sequence analysis showed that *LOC_Os05g02730* shares similarity to *PRS* in *Arabidopsis*, *NS1* and *NS2* in maize, and *OsWOX3* in rice (Dai*, et al.*^2007^, Matsumoto and Okada 2001, Nardmann*, et al.*^2004^). There are a conserved homeodomain at the N terminal and a conserved WOX motif at the C terminal of these proteins (Figure 3). Phylogenic analysis indicated that *LOC_Os05g02730* belongs to a small *NS*/*WOX3* subgroup consisting of *OsWOX3, PRS, NS1* and *NS2* (Dai*, et al.*^2007^). We sequenced and compared the 21-kb DNA fragments between markers M6 and M7 from the NIL^*GLR1*^ and NIL^*glr1*^. There is no difference in this region between NIL^*GLR1*^ and NIL^*glr1*^ plants. To understand which gene, *LOC_Os05g02720* or *LOC_Os05g02730,* is responsible for the phenotype, we analyzed their expression levels by RT-PCR. Compare to NIL^*GLR1*^, the expression level of *LOC_Os05g02720* decreased in the NIL^*glr1*^ plant (Figure 2d). However, the expression of *LOC_Os05g02730* was dramatically reduced in the NIL^*glr1*^ plant (Figure 2d). The previous studies showed that the *NS/WOX3* subgroup *WOX* genes are specifically expressed in the epidermal cells and play important roles in their differentiation (Dai*, et al.*^2007^, Ishida*, et al.*^2008^, Matsumoto and Okada 2001, Nardmann*, et al.*^2004^,Vernoud *et al.*^2009^). Therefore, *LOC_Os05g02730* is most likely the candidate gene responsible for the rice glabrous phenotype.

**Table 1:** **Molecular markers developed in this study**

Primer		Primer sequence(5′-3′)	Primer types	Genetic distance(cM)
M1	Forward	TGGTTATTTGTTATTTTAGTTGGGTG	InDell	1.38
	Reverse	TAGACTAGAGTTGGAGACG		
M2	Forward	ACGCACGCCATTACAAAC	SSR	0.44
	Reverse	CAGGAGGTGGGCCTCATT		
M3	Forward	ACGACCCACCAGCAGATA	InDell	9.43
	Reverse	AGGGACGTGAATGAAACT		
M4	Forward	GCCCTTGATCCGGTGCTCT	InDell	1.07
	Reverse	GTGTTAGATGCGTGTATT		
M5	Forward	GGGGAAGCTCATTGTCGG	InDell	0.10
	Reverse	CAGTGGTGGAGTCAAAAT		
M6	Forward	GTAGTAGTAGGAGCACAGC	InDell	0.07
	Reverse	CAATGCTGCATGGTGGTA		
M7	Forward	AACAAATCCTCCTGTTCC	CAPS	0.07
	Reverse	CGAGCTACTACTCCTGCT		
M8	Forward	ATTGCTGGCACATTTTCT	InDell	0.07
	Reverse	CATTTTCTTCCTATCTAA		
M9	Forward	CTAAGCAAGCTGACGTGTAAT	InDell	0.07
	Reverse	AACCAAATAGCACTTTCACA		
M10	Forward	TCTGTTTCGTTGGATTAGT	InDell	0.04
	Reverse	ACGAGGCATTCTTGATGG		

### Altering the expression levels of *GLR1* could partially change the glabrous phenotype

**Figure 4:**
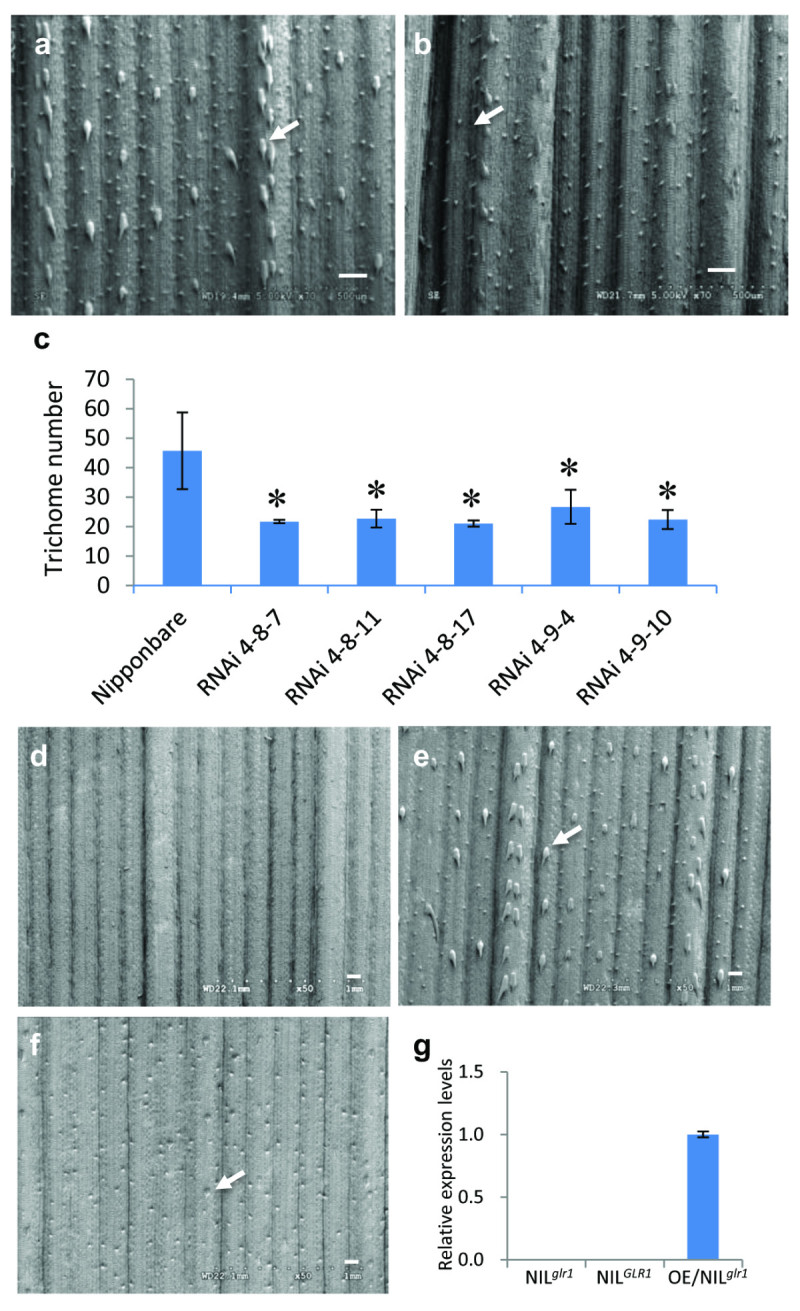
**Glabrous phenotypes of** ***GLR1*** **RNAi transgenic lines.** (**a**, **b**) The SEM images of the abaxial leaf sides of Nipponbare (**a**) and *GLR1* RNAi transgenic plants (**b**). (**c**) The macrohair number was significantly decreased in *GLR1* RNAi transgenic plants compared with that in Nipponbare (T-test, P<0.05); (**d**-**f**) The SEM images of the abaxial leaf sides of NIL^*glr1*^ (**d**), NIL^*GLR1*^ (**e**) and OE/NIL^*glr1*^ transgenic plants (**f**) ; (**g**) The relative expression levels of *LOC_Os05g02730* in leaves of NIL^*glr1*^, NIL^*GLR1*^ and OE/NIL^*glr1*^ plants. Arrows indicate the macrohairs, bar = 1 mm.

To confirm *LOC_Os05g02730* is the *GLR1* gene*,* we generated transgenic plants in a pubescence *japonica* variety Nipponbare background by the RNA interference (RNAi) method (Figure 4a). SEM analysis showed that much fewer trichomes on leaves of the RNAi transgenic lines have been observed (Figure 4b), and a further statistical analysis showed that the macrohair number on the RNAi transgenic leaves was significantly decreased (Figure 4c). When constitutively express *GLR1* in NIL^*glr1*^, it can partially rescue glabrous phenotype of NIL^*glr1*^ of T0 transgenic plants (Figure 4 d-g). These results indicate that *LOC_Os05g02730* is the gene responsible for the glabrous phenotype of the NIL^*glr1*^ plant.

### DNA methylation may be involved in the expression of *GLR1*

**Figure 5:**
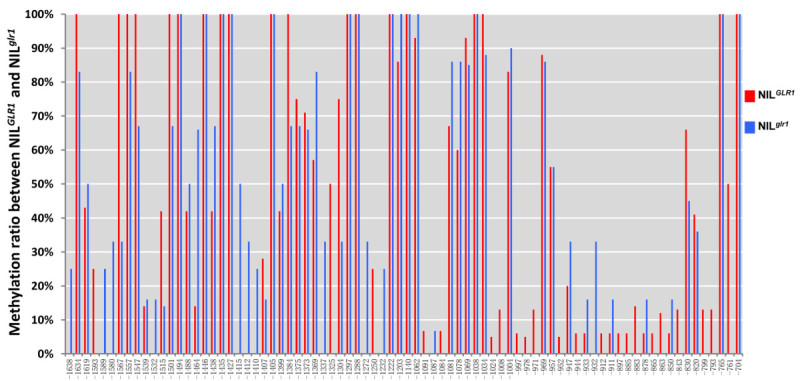
**Comparison of the DNA methylation between NIL**^***GLR1***^**and NIL** ^***glr1***^**.** DNA methylation levels (%) of the *GLR1* and *glr1* DNA sequences of the NIL^*GLR1*^ (red) and NIL^*glr1*^ (blue) plants are analyzed. The numbers indicate the position in the 2-kb upstream region starting from the start codon.

The findings that no mutation was found in the *GLR1*-containing mapping region and that the expression of *LOC_Os05g02730* was unable to be detected in the NIL^*glr1*^ plant strongly suggests that *GLR1* may be regulated epigenetically through a DNA methylation mechanism. We therefore carried out a bisulfite sequencing experiment to examine whether DNA methylation are involved in the regulation of *GLR1*. As shown in Figure 5, the bisulfite sequencing of the 2.0-kb promoter region of *GLR1* revealed some apparent methylation differences between NIL^*glr1*^ and NIL^*GLR1*^, suggesting that an epigenetic mechanism may involve in the regulation of the *GLR1* expression.

## Discussion

In *Arabidopsis*, trichomes have been served as an excellent model system to study plant cell differentiation (Ishida et al. 2008). Glabrous mutants that are defective in leaf hair or trichome have been identified in many plant species. However, genes controlling trichome development in rice have not been identified up to date yet. *GL1* was previously mapped on the short arm of chromosome 5 (Li, et al. 2010, Wang et al. 2009, Yu et al. 1995) and it was proposed that a single nucleotide mutation (A to T) in the 5’UTR of *LOC_Os05g02754* (*Os05g0118900*), which encode an unknown protein, might be responsible for the *gl1* trait (Li, et al. 2010). Our mapping data suggested that *glr1* may be allelic to *gl1*. We compared the sequences of 5’UTR of *LOC_Os05g02754* (*Os05g0118900*) of NIL^*GLR1*^ and NIL^*glr1*^ and found that the indicated position of 5’ UTR of *LOC_Os05g02754* (*Os05g0118900*) in the NIL^*GLR1*^ plant is A and that in the NIL^*glr1*^ plant is T. However, our data indicate that instead of *LOC_Os05g02754* (*Os05g0118900*) *LOC_Os05g02730* (*Os05g0118700*), which encode a WUS-like homeodomain protein, controls the glabrous phenotype. First, the mapping data has pinpointed the *GLR1* locus within a 21-kb region that contains only two predicted genes, *LOC_Os05g02720* (*Os05g0118600*) and *LOC_Os05g02730* (*Os05g0118700*). Second, comparison of the gene expression levels of *LOC_Os05g02720* and *LOC_Os05g02730* between NIL^*GLR1*^ and NIL^*glr1*^ plants showed that *LOC_Os05g02730* is dramatically increased in the NIL^*glr1*^ plant, however, change of *LOC_Os05g02720* in the NIL^*glr1*^ plant is not very significant. In contrast, no difference of expression levels of *LOC_Os05g02754* has been detected between NIL^*GLR1*^ and NIL^*glr1*^ plants (Figure 2d). Third, an apparent decrease in the trichome number on leaves and glumes of *GLR1* RNAi transgenic plants have been obtained. Overexpression of *GLR1* in NIL^*glr*^ partially rescues glabrous phenotype of the NIL^*glr1*^ plant. Fourth, the sequence alignment showed that *GLR1* has high similarity to previously identified homeodomain proteins, whose functions are essential for the differentiation of epidermal cells (Dai, et al. 2007, Matsumoto and Okada 2001, Nardmann, et al. 2004). Taken all these together, we strongly suggest that the WUS-like homeodomain protein encoded by *LOC_Os05g02730* is the *GLR1* gene and is essential for the trichome development in rice.

The RNAi of *GLR1* reduced the trichome number in transgenic lines, while the trichome is almost completely lost in NIL^*glr1*^. This discrepancy could result from the different genetic background of plant materials or from incomplete suppression of the target gene in transgenic plants. Alternatively, the expression *LOC_Os05g02720* is also decreased in the NIL^*glr1*^ plant*,* which implies that *LOC_Os05g02720* may be also involve in trichome development, thus knockdown of *LOC_Os05g02730* alone cannot completely suppress the trichome development. Further knockdown *LOC_Os05g02720* along or knockdown it together with *LOC_Os05g02730* will clarify the role of *LOC_Os05g02720*.

Chromatin state controls gene expression and plays critical roles in development. In plant, trimethylated K9 of histone H3 (H3K9me3) indicates an open chromatin state, while monomethylated and dimethylated H3K9s (H3K9me1 and H3K9me2) indicate a closed state (Liu et al. 2010). The identification of GL2 EXPRESSION MODULATOR (GEM) indicates that the regulation of *GL2* expression is more complicated than previously expected (Caro, et al. 2007). Trichome density increased in *gem-1* mutant whereas decreased in *GEM*-overexpressing plants. Consistent with the phenotype, the *GL2* expression has been observed to increase in *gem-1* whereas decrease in *GEM*-overexpressing plants (Caro, et al. 2007). It has been observed that H3K9me3 increases and H3K9me2 decreases in the *GL2* promoter in the *gem-1* background, but H3K9me3 decreases and H3K9me2 increases in *GEM*-overexpressing plants (Caro, et al. 2007). This kind of epigenetic control may also be involved in rice trichome development. Rice SET Domain Group Protein 714 (SDG714) functions as a histone H3K9 methyltransferase, which is involved in histone H3K9 methylation, DNA methylation and genome stability (Ding et al. 2007). Loss of macrohairs but not microhairs on leaves of the SDG714 RNAi transgenic plants indicated that regulation of chromatin status of some unidentified regulators may play an important role in the trichome development in rice (Ding *et al.*^2007^). In agreeable to these findings, the genomic bisulfite sequencing of *GLR1* showed that the DNA methylation pattern at several sites of the *GLR1* promoter region in the NIL^*glr1*^ plants is different from that in the NIL^*GLR 1*^ plants, though no sequence difference of *GLR1* was found between the NIL^*GLR 1*^ and NIL^*glr1*^ plants. These results indicated that the epigenetic mechanism may be involved in the regulation of the *GLR1* expression and the trichome development in rice. Although, different patterns of the DNA methylation in upstream region of *LOC_Os05g02730* (*Os05g0118700*) between NIL^*GLR1*^ and NIL^*glr1*^ has been observed, we are unable to determine which sites are responsible for suppression of *LOC_Os05g02730*. Moreover, the *GLR1* expression driven by a constitutive promoter dramatically increased the expression of *GLR1,* but cannot completely rescue the glabrous phenotype in T0 transgenic plants (Figure 4 d-g). It indicates that the regulation of the *GLR1* expression and the trichome development in rice is more complicate than expected. Further investigation is needed to uncover the molecular mechanism of *GLR1* expression regulation.

Glabrous rice varieties are widely cultivated in America and Africa, while most varieties cultivated in Asia are pubescent (Khush*, et al.*^2001^). In higher plants, although trichomes are thought to be important for plant defense against biotic and abiotic stresses, glabrous trait may be a selectively neutral trait in rice. Previous studies have indicated that the introduction of glabrous trait into *japonica* varieties may not cause any obvious disadvantages in plant defense (Li *et al.*^2011^). In agriculture, the interest of breeding glabrous elite rice varieties is mainly due to its practical advantages of greater packing capability and less itching effect during the harvest process. The cloning of *GLR1* will not only help to understand the molecular mechanism of trichome development in rice but also improve the efficiency of breeding glabrous elite rice varieties by marker-assisted selection and genetic modification approaches.

## Conclusions

*GLR1* plays an important role in rice trichome development and will contribute to breeding of glabrous elite rice varieties

## Methods

### Plant materials

Jia64 is a glabrous variety derived from American rice variety Rico No.1 and Jia33 is a pubescent variety in southeast China. Rice plants were cultivated in the experimental field of Jiaxing Academy of Agricultural Science in growing seasons from May to October.

### Scanning electron microscopy

Samples were prepared as described previously (Li *et al.*^2009^). Briefly, samples were fixed with 2.5% (v/v) glutaraldehyde in 0.1 M sodium phosphate buffer (PBS, pH 7.2) at 4°C overnight. After being rinsed with 0.1 M PBS twice, samples were post-fixed in 1% (w/v) osmium tetroxide for 2 h at 4°C. Samples were rinsed with the same buffer for 2 more times and then dehydrated in a graded series of ethanol. For scanning electron microscopy, samples were critical-point dried (Hitachi HCP-2) and observed under a scanning electron microscope (Hitachi S-3000N).

### Genetic mapping of *GLR1*

An F_2_ mapping population was generated from a cross between Jia64 and Jia33. 24 molecular markers were used for genetic linkage analysis of 44 F_2_ plants that show the glabrous phenotype. To fine-map *GLR1*, new PCR-based markers were developed and 1,447 F_2_ glabrous plants were analyzed using markers as given in Table 1. The *GLR1* locus was further narrowed within an interval of 21-kb DNA fragment between the M6 and M7 markers. To sequence the *GLR1* locus, the entire genomic region was amplified from NIL^*GLR1*^ and NIL^*glr1*^ by PCR with LA-Taq (TaKaRa).

### RNA extraction and reverse transcription-polymerase chain reaction (RT-PCR)

Total RNA was isolated from rice plants by Trizol extraction method (Invitrogen Life Technologies). To conduct RT-PCR analyses, cDNA strands are synthesized by the SuperScript III RT kit (Invitrogen Life Technologies). Real-time PCR analysis were performed using the SYBR Green RT-PCR kit (Biorad). Primers RT1-F and RT1-R were used to amplify *LOC_Os05g02720,* RT2-F and RT2-R to *LOC_Os05g02730* and primers RT3-F and RT3-R to *LOC_Os05g02754* (Table 2).

**Table 2:** **Primers for RT-PCR and RNAi construct**

Primer	Primer sequence (5′-3′)
RT1-F	GGCAAGGCATCAGTTAGTG
RT1-R	GCCAGAGGTTCCTTCCAA
RT2-F	GCCGCAGCAGCAGCAGCAGCAGCTTACA
RT2-R	TCCACTAGCTTCCCCAGCGAGTAGTCCG
RT3-F	GTCCTCCCTCAGCTTCTTCATCGTCA
RT3-R	GAAGCACATCGCCGCCGTCTCC
RNAi1-1f	ACGGATCCTTAATCATTGCTTAATCGATCA
RNAi1-1r	GAGGTACCCGTCATGCTGCTCTTCCT
RNAi1-2f	GGAGCTCTTAATCATTGCTTAATCGATCA
RNAi1-2r	GCACTAGTCGTCATGCTGCTCTTCCT

### Plasmid construction and rice transformation

To generate the RNAi construct, two DNA fragments RNAi 1-1 and RNAi 1-2 were amplified respectively by primers RNAi 1-1f and RNAi1-1r, RNAi1-2f and RNAi1-2r (Table 2). The construct 1460-RNAi 1-1 was generated by digesting the RNAi 1-1 fragment with *Bam* H I and *Kpn* I and ligated to the binary vector 1460 by T4 DNA ligases. The hairpin cassette was generated by digesting the RNAi 1-2 fragment with *Sac* I and *Spe* I and ligated in reversed direction of fragment RNAi 1-1 to construct 1460-RNAi 1-1. For construction of the overexpression cassette, the coding region of *GLR1* was amplified and liagated to the 1460 vector by BamH I and Spe I. The constructs were confirmed by sequencing and introduced into *Agrobacterium tumefaciens* strain EHA105 by electroporation. The rice (Nipponbare) transformation was performed as described previously (Hiei *et al.*^1994^). For RNAi transgenic plants, T2 lines derived from individual transgenic lines were used for further analysis. T2 Lines RNAi 4-8-7, RNAi 4-8-11 and RNAi 4-8-17 were derived from line RNAi 4-8. T2 Lines RNAi 4-9-4 and RNAi 4-9-11 were derived from line RNAi 4-9. For overexpression transgenic plants, T0 plants were used for analysis.

### Bisulfite sequencing

Genomic DNA extracted by the CTAB method and 1.0 μg DNA was bisulfite treated using the Bisulfite kit (Qiagen 59104). The candidate 2-kb upstream of the coding region of *LOC_Os05g02730* was amplified using listed bisulfite primers (Table 3). The PCR products were cloned into the pGEM-T easy vector (Promega) for sequencing.

**Table 3:** **Primers for Bisulfite sequencing**

Primer	Primer sequence(5′-3′)
ME-3F	AGAGTTTGTTATGGGTGTAATGTTTGTTTA
MEQ-3F	GATTTGTTATTATAGGTATTATAAAGAGA
ME-3R	TTACATCTCATAAAAATATTTTATTAACT
MEQ-3R	TAAAAAACAAACACCTATCCCTATCCTAC
ME-4F	TTTTTGAGAATTATTAGATTTTTTTATGGT
MEQ-4F	TTGTTTTTTTATTAATTATTTTATTAAT
ME-4R	AATACTAATAAACAATACATCAATCCTCTT
MEQ-4R	CCACAATAAAACATAAAAATCACAAAACTA
ME-5F	AATATAATGGATTATTTGGTGGATTAGTTT
MEQ-5F	GGTAATTTTTTTTTTTTATTTTTAGTGTTA
ME-5R	ACATAACACACTAAAACAAAAAATTCATA
MEQ-5R	CTTTTACATCATCACTATATAATAACAATT
ME-6F	GGTTATTTTGGATTATGTTAATATGTTAGG
MEQ-6F	TTTAAGTTGGTAGTTTTTTTTGGTTTTTAG
ME-7F	ATATTATATTAGATGTGGGAGTATTAATT
MEQ-7F	AGGTTATTTTGGATTATGTTAATATGTTAG
ME-7R	ATATAACTATTATTTAATTAATACCTAACT
MEQ-7R	TAAATATAATTACTTCCAATCAATTAAA
ME-8F	TTGAATAAAATATGTAGTAATATGTTTATT
MEQ-8F	TTTGTATATTTTGGGGTGGTAATTTTATT
ME-8R	ATCAACCCCACCACCACCACCCCCATCAA
MEQ-8R	TCCACCACCACCACCACTCTACTACTACTA
ME-9F	ATATAAGAAAATTTAGTTATTTAGGTAG
MEQ-9F	ATAGGAGGAGGGATATATGGTGTTGGTGGT
ME-9R	AAAAATAATAAAAAATACAAAAACAACAA
MEQ-9R	CAAAACTTCTAACAATCACAAACCTTATA

## Authors’ original submitted files for images

Below are the links to the authors’ original submitted files for images. Authors’ original file for figure 1 Authors’ original file for figure 2 Authors’ original file for figure 3 Authors’ original file for figure 4 Authors’ original file for figure 5
