# Supplementary figures and images for: Glabrous Rice 1, encoding a homeodomain protein, regulates trichome development in rice
Source: Rice (N Y). 2012 Oct 6;5:32. doi: 10.1186/1939-8433-5-32 (PMC4883694; doi:10.1186/1939-8433-5-32)

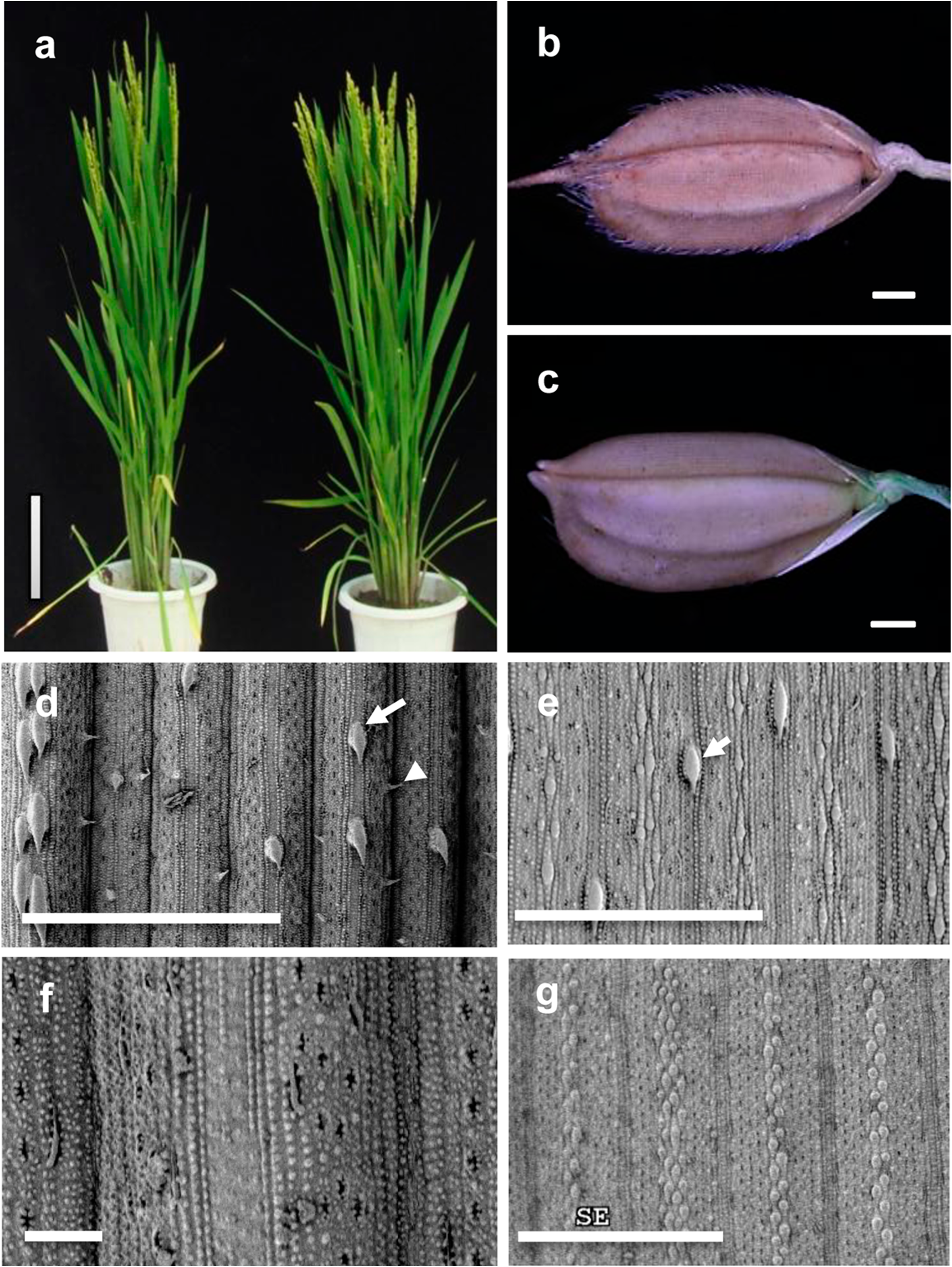

Supplement: Supplementary file 1 — Authors’ original file for figure 1 [file 12284_2012_29_MOESM1_ESM.tiff]

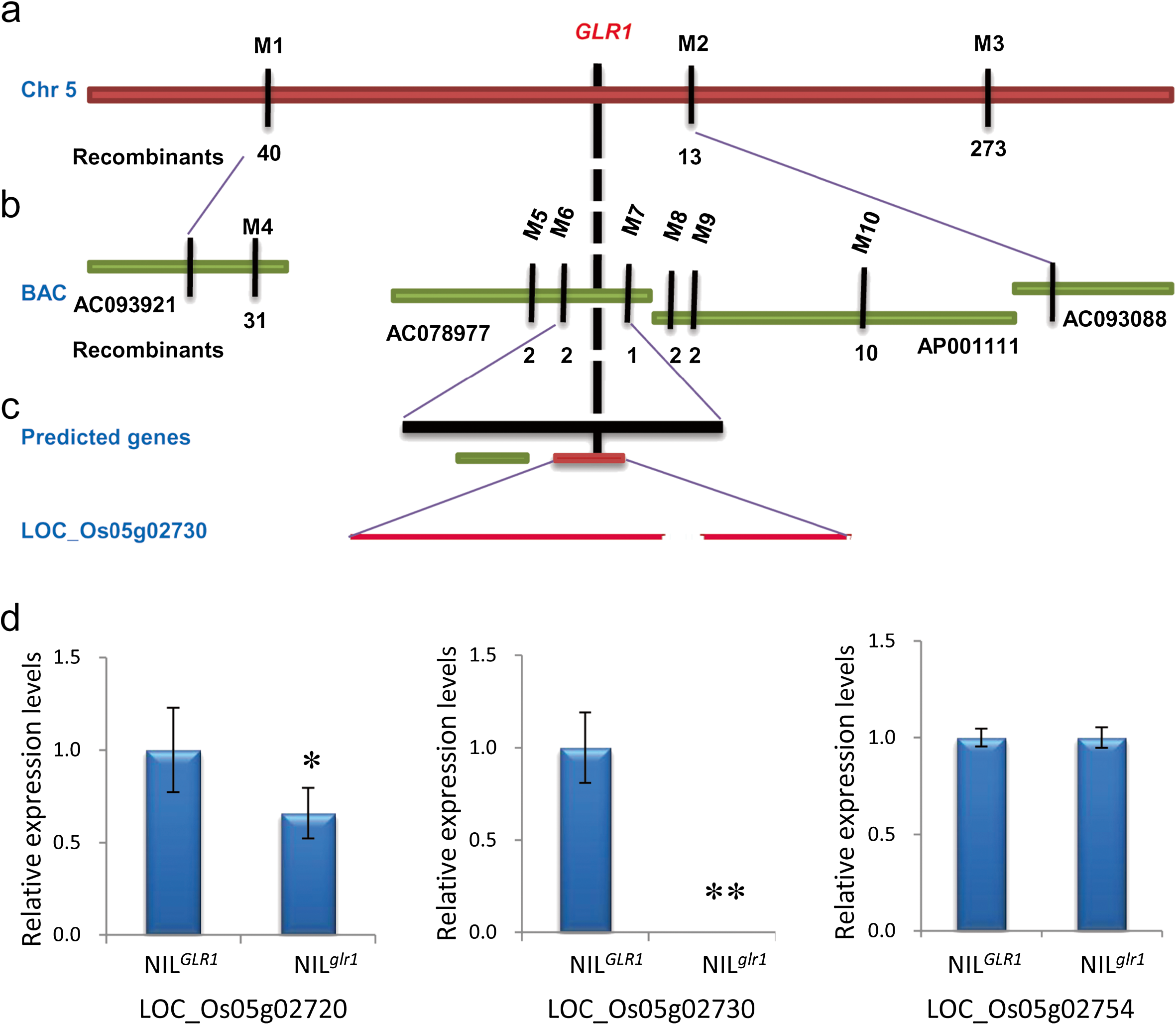

Supplement: Supplementary file 2 — Authors’ original file for figure 2 [file 12284_2012_29_MOESM2_ESM.tiff]

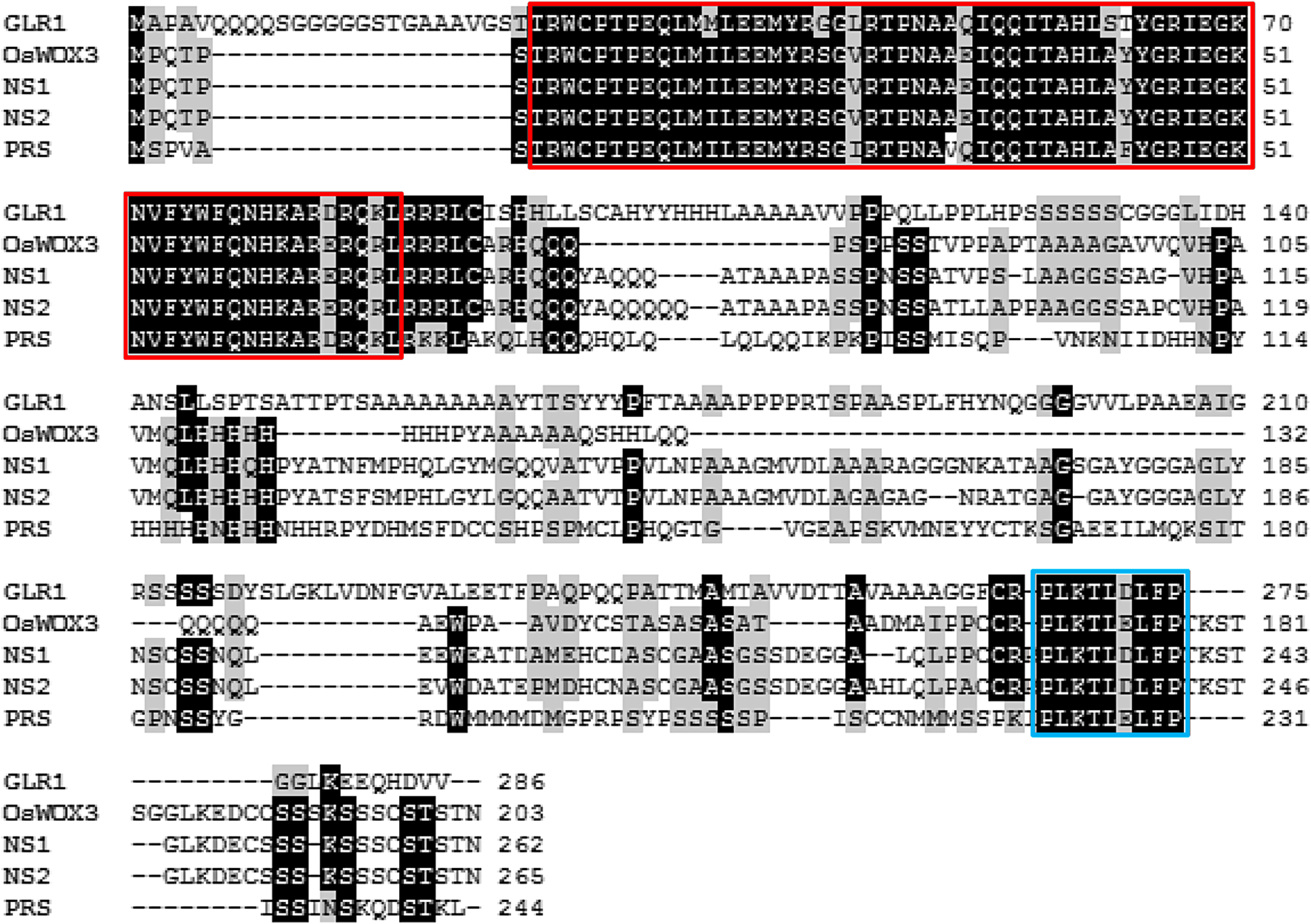

Supplement: Supplementary file 3 — Authors’ original file for figure 3 [file 12284_2012_29_MOESM3_ESM.tiff]

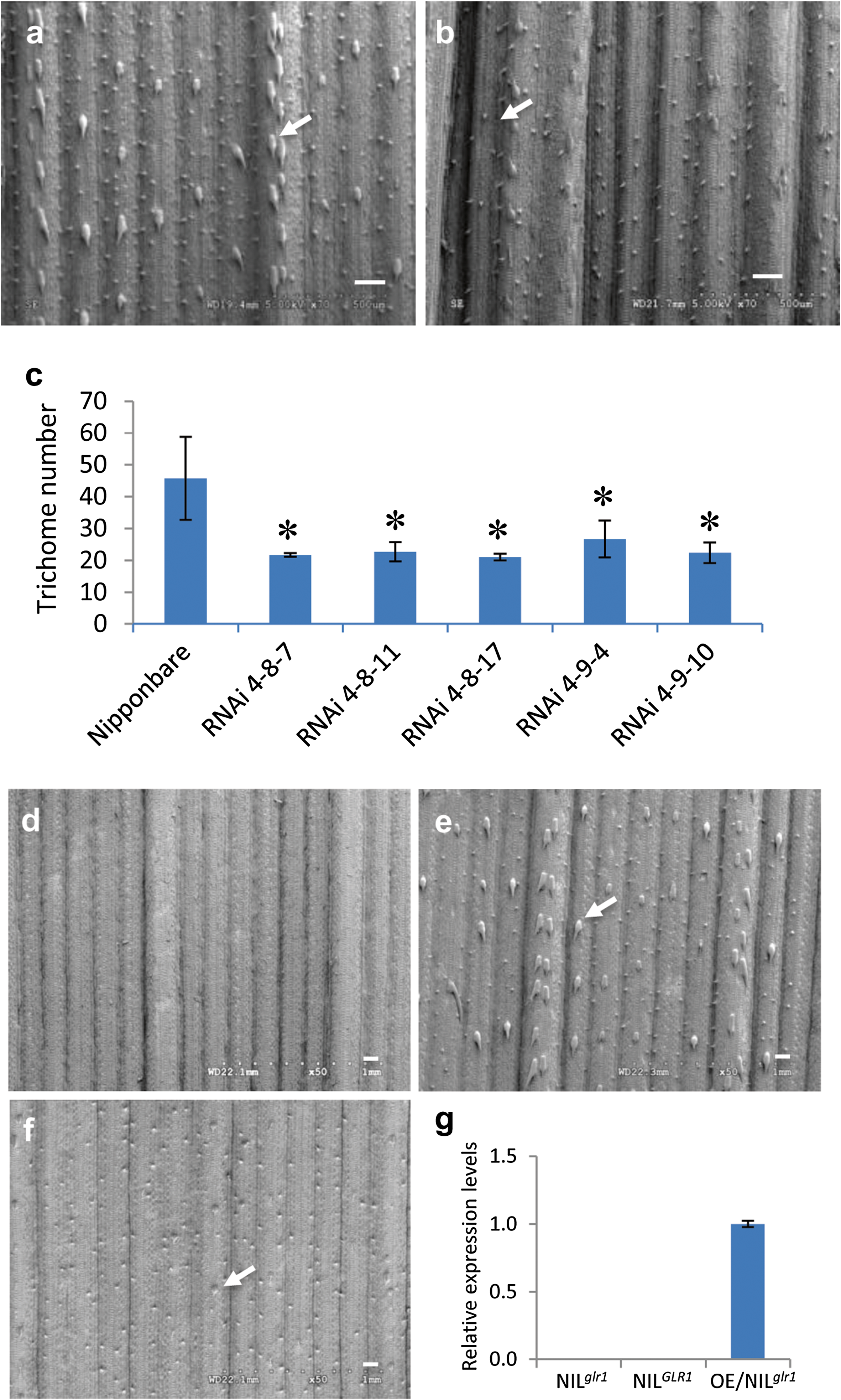

Supplement: Supplementary file 4 — Authors’ original file for figure 4 [file 12284_2012_29_MOESM4_ESM.tiff]

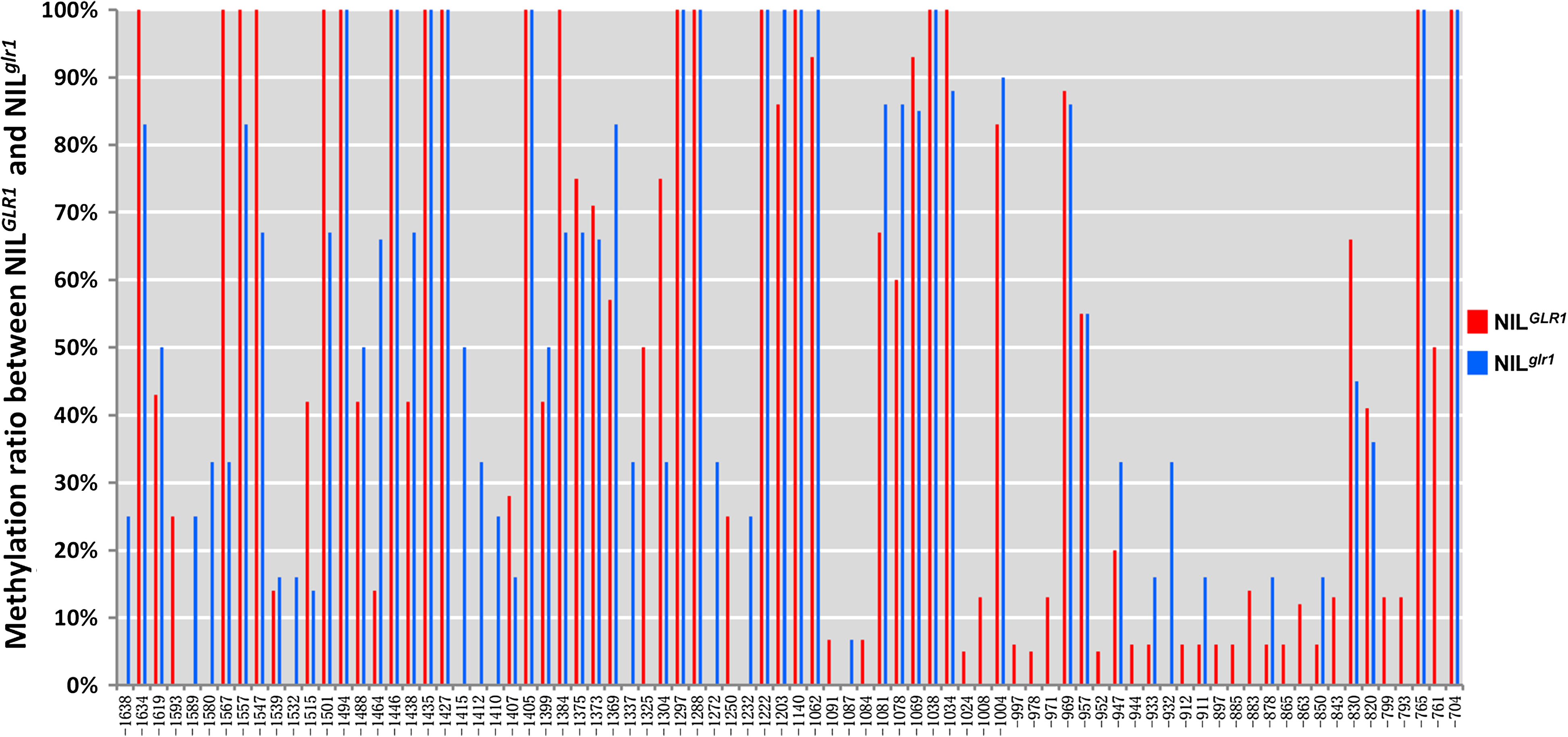

Supplement: Supplementary file 5 — Authors’ original file for figure 5 [file 12284_2012_29_MOESM5_ESM.tiff]
